# Review on oral mucosal lesions in HIV patients with and without highly HAART

**DOI:** 10.6026/9732063002001629

**Published:** 2024-11-30

**Authors:** Nishath Sayed Abdul, Moaath Ahmad Alsayegh, Eida Ahmed Shahin, Ra'ed Ghaleb Salma, Kholoud Abdullah Alamri

**Affiliations:** 1 Department of OMFS and Diagnostic Sciences, College of Medicine and Dentistry Riyadh Elm University, Riyadh, Kingdom of Saudi Arabia; 2 Department of Oral Medicine & Diagnostic Sciences, College of Medicine and Dentistry, Riyadh Elm University, Riyadh, Kingdom of Saudi Arabia; 3 Prince Sultan Military Medical City, Riyadh, Kingdom of Saudi Arabia

**Keywords:** Oral mucosal lesions, HIV, Highly active antiretroviral therapy, CD4 count

## Abstract

This review was hence conducted to understand the prevalence and types of OMLs in individuals with HIV and the influence of highly active antiretroviral therapy (HAART) on these manifestations. A comprehensive search was conducted across multiple databases, namely PubMed, Web of Science, Scopus, EMBASE, CINAHL, Cochrane Library, ProQuest and Google Scholar. Two reviewers independently extracted the data variables. Studies included had a lower risk of bias as per the NOS assessment. The findings revealed a significant prevalence of OMLs among individuals with HIV, with variations in the occurrence and type of these OMLs between individuals on HAART and those not on HART. Overall, it was noted that the occurrence of OMLs was lessened with HAART treatment as compared to those without.

## Background:

The recent COVID-19 pandemic has emphasised that communicable and infectious diseases still pose a threat to the health of the common population. Our preparedness for a pandemic of this scale and magnitude is still by far not up to satisfactory levels; however, there are also other major communicable ailments that have been wreaking havoc for decades, the most fatal of them being HIV/AIDS [1]. With over 38 million people living with the disease worldwide, this viral disease continues to remain a significant challenge to researchers and healthcare personnel globally [2]. Oral mucosal lesions (OMLs), an umbrella term for a range of conditions including oral candidiasis, oral hairy leukoplakia and oral Kaposi's sarcoma, have been observed to be common in HIV/AIDS-affected patients [3, 4- 5]. These lesions can significantly compromise the quality of life, causing discomfort, pain and difficulties in eating and speaking, as well as the subsequent quality of life [6]. Furthermore, the presence of OMLs can often be an early indicator of a declining immune status [7]. OMLs also tend to play an important diagnostic role in nearly all systemic diseases that tend to plague healthcare facilities around the world [8]. Apart from this, they also tend to play a pivotal role in the identification of non-systemic diseases [9].

Highly active antiretroviral therapy (HAART), a combination of at least three antiretroviral drugs, has been a game changer in the management of HIV/AIDS since its introduction in the mid-1990s. Over the ensuing years, this therapeutic approach has involved a repertoire of over 30 distinct pharmaceutical agents, classified into six unique categories [10]. Each of these drug groups offers its own set of benefits and limitations. One of the fundamental objectives of HAART is to inhibit viral replication, thereby creating an environment conducive to the restoration of immune functions. This in turn helps in disease progression and reducing the incidence of opportunistic infections [11]. However, the impact of HAART on the prevalence and severity of OMLs in HIV/AIDS patients remains a subject of on-going research; with studies reporting varying findings [12-13], since these therapeutic agents also have a potential downside as they may diminish the resistance of the virus to the drugs. Consequently, HAART has been associated with an elevation in the counts of CD4+ T lymphocytes and a reduction in the viral load among HIV-infected individuals [12- 13]. Despite these immunological improvements, patients undergoing HAART have been observed to experience clinical episodes of infectious diseases. This suggests a complex interplay between immune reconstitution and disease susceptibility [13]. Therefore, this systematic review aims to compare the occurrence of OMLs in HIV/AIDS patients on HAART with those not on HAART.

## Methodology:

## PRISMA protocol:

The current systematic review followed the PRISMA (Preferred Reporting Items for Systematic Reviews and Meta-Analyses) protocol [14], as displayed in Figure 1 (see PDF). The study was registered at the Riyadh Elm University (REU) Research Centre and obtained the registration number "FPGRP/2024/864."

## PECO protocol:

The PECO protocol followed for this study is listed below:

[1] Population (P): Human individuals diagnosed with HIV/AIDS of all age groups and genders

[2] Intervention (I): Administration of HAART to the affected population

[3] Comparator (C): HIV/AIDS patients who are not on HAART

[4] Outcome (O): Occurrence of OMLs in terms of prevalence, variation and severity.

## Search strategy:

The databases utilised for the selection of relevant studies for this review were PubMed, Web of Science, Scopus, EMBASE, CINAHL, Cochrane Library, Pro-Quest and Google Scholar. The search technique was modified using MeSH terms and Boolean operators, as shown in Table 1.

## Selection criterion:

For inclusion, the studies had to meet the following criteria: (1) Clinical studies/articles providing clear information about the HAART regimen and which reported on the occurrence of oral mucosal lesions (OMLs) (2) Published in English language; (3) Articles providing comparative data between groups on HAART and those not on HAART. Articles were excluded if they were: (1) reviews, case reports, commentaries, editorials, or letters to the editor; (2) not published in English; (3) focused on animal or in vitro studies; (4) did not provide clear information about the HAART regimen or lacked data on patients who were not on HAART; (5) did not specifically report on the occurrence of OMLs; and (6) did not provide comparative data between groups on HAART and those who were not.

## Data extraction:

A predesigned data extraction form was used to ensure consistency across all studies. The data extraction form included the following information fields: (1) author(s) and year of publication; (2) sample size; (3) demographic characteristics of the participants; (4) HAART regimen; (5) evaluation method for OMLs; and (6) key findings related to the comparison of OMLs in patients on HAART and those not on HAART. Two independent reviewers were assigned to each study. They independently extracted the data and compared their findings. Any discrepancies between the two reviewers were resolved through discussion until consensus was reached. If consensus could not be reached, a third reviewer was consulted.

## Bias assessment protocol:

The assessment of potential biases within the included studies for this systematic review was performed using the Newcastle-Ottawa Scale (NOS) [15]. The NOS assesses the quality of studies based on three broad categories: selection of study groups (0-4 points), comparability of study groups (0-2 points) and assessment of outcomes (0-3 points). The studies are graded on a scale from 0 to 9. Studies with a total score of 7-9 are considered to have a low risk of bias; scores of 4-6 indicate a moderate risk of bias; and scores of 0-3 indicate a high risk of bias.

## Results:

## Study selection:

Initially, a comprehensive search was conducted across multiple databases, yielding a total of 471 records. No records were identified from the registers. Before the formal screening process, certain records were excluded. These included 69 reviews, 78 case reports and editorials and 31 non-English papers. After these exclusions, the total number of records was reduced to 293, which were subsequently screened. Duplicate records were identified and excluded, resulting in the removal of 49 additional studies. The remaining 244 studies were then assessed for retrieval and a total of 182 reports were selected for further inspection. However, 36 of these reports were not retrieved, reducing the number to 146. Further exclusions were made based on the relevance of the reports to the PECO framework as well as the overall topic of the review. This resulted in the exclusion of an additional 85 reports (47 not responding to PECO and 38 being off-topic), leaving 61 reports for eligibility assessment. In the end, nine studies [16, 17, 18, 19, 20, 21, 22, 23-24] met all the inclusion criteria and were selected for this review (Figure 1 see PDF).

## Study characteristics:

The demographic and inferential assessments of the included papers [16, 17, 18, 19, 20, 21, 22, 23-24] are represented through Table 2 and Table 3, respectively. The studies were conducted across various regions, including Africa [16, 17, 18], Asia [19, 20, 21, 22, 23] and Europe [24]. The research studies comprised a diverse range of sample sizes, varying from as few as 81 participants to as many as 1812 participants [19, 20, 21, 22, 23, 24]. The mean age of participants varied widely, with the lowest mean age recorded in the Tanzanian study at 7.6 ± 4.3 years and the highest mean age not explicitly stated but up to 67 years in the Tanzanian study [16, 17, 18]. The assessment periods varied markedly between studies, ranging from 5 months to over 36 months, with some studies not specifying the assessment duration [16, 17, 18, 19, 20, 21, 22, 23-24].

## Main findings:

Overall, all nine studies showed that oral mucosal lesions were significantly less common in the HAART group as compared to the non-HAART category. Candidiasis was the most prevalent oral lesions, present in all studies. E.C. cleaning house categorization and WHO category were used for oral mucosal assessment.

## Risk of bias:

Overall, all the studies showed a low risk of bias, as assessed by the New Castle Ottawa Scale. The schematics are shown in Table 4.

## Discussion:

The systematic review suggested that HAART plays a significant role in the prevalence and type of oral lesions in individuals with HIV. However, due to the differences in the results across studies, further research is necessary to establish more precise associations and causality between HAART and specific OMLs. The variations in the findings could be due to differences in the study populations, the duration of HAART, or other factors not controlled for in the studies. Oral candidiasis was the most common lesion observed across the studies. Hamza *et al.* [16] found no oral candidiasis in individuals on HAART, while individuals not on HAART exhibited a prevalence of 20.7%. Similarly, Naidu *et al.* [19] reported that the prevalence of pseudomembranous candidiasis and erythematous candidiasis was higher in patients not on HAART. This trend of a higher prevalence of candidiasis in patients not on HAART was also observed by Patil *et al.* [21] and Tappuni *et al.* [25]. Oral hyperpigmentation and oral hairy leukopenia were other common lesions observed. Mthethwa *et al.* [18] found a high prevalence of hyperpigmentation and oral hairy leukoplakia among individuals undergoing HAART. However, Rai *et al.* [22] noted a higher incidence of hyperpigmentation in the HAART group than in the non-HAART group, suggesting that HAART may be associated with increased melanosis. Interestingly, while some lesions were more prevalent among individuals on HAART, others were more common among individuals not on HAART. Shu *et al.* [23] found a higher prevalence of erythematous candidiasis and pseudomembranous candidiasis among non-ART patients, while oral hairy leukoplakia (OHL), necrotising ulcerative gingivitis (NUG) and necrotising ulcerative periodontitis (NUP) were found more frequently in the ART group. In our review, we observed a significant prevalence of OMLs among HIV-positive individuals, with variations in the prevalence and types of OMLs between those on HART and those not on HART. This echoes the findings of the study by De Almeida *et al.* [25], which also indicated a lower prevalence of certain oral lesions, including angular cheilitis and herpes, in patients on HAART.

Lauritano *et al.* [26] focused on the impact of oral hard and soft tissue lesions on the quality of life of HIV-positive paediatric patients. Their study found that candidiasis was the most prevalent oral lesion in HIV-positive children, which aligns with our findings in the adult population. Ottir *et al.*'s study [27] assessed the correlation between HIV-related oral manifestations, HAART and CD4+ T-cell count. Their findings are in accordance with ours concerning the decline in the incidence of certain oral lesions, such as oral candidiasis, oral hairy leukoplakia and Kaposi's sarcoma, following the introduction of HAART. However, they noted a significant correlation only between an increase in CD4+ T-cell count and a decrease in oral candidiasis, which provides an interesting perspective on our own findings. The results of our review align with the findings of Araujo *et al.* [28], who also identified a reduction in the prevalence of oral manifestations in HIV-positive paediatric patients undergoing HAART compared to those on antiretroviral therapy (ART). The common oral lesions identified in both studies were oral candidiasis and gingivitis. Araujo *et al.* also reported parotid gland enlargement and linear gingival erythema, indicating possible age-related variations in oral manifestations among HIV-positive individuals. Varadarajan *et al.* [29] focused on oral pigmented lesions in individuals with HIV and the impact of HAART on these manifestations. While our review did not specifically focus on pigmented lesions, we observed a higher prevalence of oral mucosal hyperpigmentation among individuals on HART, reflecting similarities in the findings. Varadarajan *et al.* also discussed the reduction in HIV-associated mortality rates following the introduction of HAART, which, although not a direct focus of our review, supports our findings on the importance of HAART in managing oral health and overall survival in HIV-positive individuals. The therapeutic efficacy of HAART in HIV-positive patients is more accurately evaluated when considering both the CD4 count and the CD4/CD8 ratio. This approach has been supported by multiple studies, which highlight the CD4/CD8 ratio as an immuno-stimulatory biomarker indicative of non-AIDS morbidity and chronic inflammation [30, 31, 32- 34]. In patients with untreated HIV infection, a rise in CD8 cell counts accompanies a decline in CD4+ cell counts [35]. International clinical data suggests that various oral lesions become apparent in the initial 1-4 years preceding the onset of AIDS, likely linked to a diminished CD4 count [36]. Numerous studies have identified a reverse association between the CD4 cell count and the prevalence of oral lesions in HIV-positive patients, where a lower CD4 count (<200/µl) is linked with a higher incidence of oral lesions [21, 23-24]. Rao *et al.*'s study reported a high incidence of periodontal disease in patients, followed by hyperpigmentation [37]. However, it should be noted that while some studies found a lower CD4 cell count associated with a higher prevalence of oral lesions, others found that a higher CD4 count exacerbated clinical symptoms alongside oral lesions [38]. This suggests that while the CD4 count is a significant predictive factor for disease progression, it does not consistently correlate with the development or remission of oral lesions [39]. Yet another study [40] reported that the majority (51.32%) of the patients undergoing HAART showed some form of oral lesions. of which periodontitis was found in 30.77%, followed by mucosal hyperpigmentation (17.44%) and acute gingivitis (10.77%). Other conditions included oral candidiasis, linear gingival erythema, stomatitis and nonspecific ulcers. These findings suggest that a significant segment of HIV patients on HAART develop oral conditions, likely as a side effect of the antiretroviral therapy.

Based on the findings of the review, it was observed that OMLs were prevalent in individuals with HIV and the prevalence and type of these lesions varied significantly between individuals on HAART and those not on HAART. This highlighted the crucial role of HAART in the oral health of individuals with HIV. On the basis of these observations, it can be recommended that routine oral health assessments be integrated into the standard care for individuals with HIV, given the high prevalence of OMLs in this population. Regular oral examinations would facilitate early detection and treatment of OMLs, potentially improving the oral health and overall quality of life for these individuals. The significant association between HAART and the prevalence and type of OMLs also indicates a need for continued research into the impact of different antiretroviral regimens on oral health. Understanding these relationships could inform the development of tailored oral care strategies for individuals on specific antiretroviral regimens. Despite the comprehensive nature of the review and its significant findings, several limitations were noted that could impact the interpretation and generalisability of the results. There was a substantial degree of heterogeneity in the demographic characteristics, methodologies and reporting standards across the reviewed studies. This heterogeneity complicated the comparison and synthesis of findings and may have introduced an element of bias into the results. Also, some studies did not specify key information, such as the gender ratio, mean age and assessment period. The lack of these critical data points impeded a complete understanding of the factors influencing the prevalence and type of OMLs in individuals with HIV, potentially masking important associations.

## Conclusion:

The results of the review demonstrated a significant prevalence of OMLs with notable variations in the occurrence and type between individuals on HAART and those who were not. Certain OMLs, such as oral candidiasis, were found to be more prevalent among individuals not on HART, while others, including oral hyperpigmentation and oral hairy leukoplakia, showed a higher prevalence among those on HART. These findings highlight the importance of HAART in the oral health management of individuals with HIV. The review also stressed the need for routine oral health assessments to be incorporated into the standard of care for these individuals, given the high prevalence of OMLs.

## Figures and Tables

**Table 1:** Search strings employed for the database search

**Database**	**Search String**
PubMed	(("HIV"[MeSH] OR "AIDS"[MeSH]) AND ("oral mucosal lesions"[MeSH] OR "OML"[Title/Abstract]) AND ("humans"[MeSH])) AND (("antiretroviral therapy, highly active"[MeSH] OR "HAART"[Title/Abstract]) NOT ("antiretroviral therapy, highly active"[MeSH] OR "HAART"[Title/Abstract]))
Web of Science	(("HIV" OR "AIDS") AND ("oral mucosal lesions" OR "OML") AND "humans") AND (("HAART" OR "antiretroviral therapy, highly active") NOT ("HAART" OR "antiretroviral therapy, highly active"))
Scopus	((TITLE-ABS-KEY("HIV") OR TITLE-ABS-KEY("AIDS")) AND (TITLE-ABS-KEY("oral mucosal lesions") OR TITLE-ABS-KEY("OML")) AND TITLE-ABS-KEY("humans")) AND ((TITLE-ABS-KEY("HAART") OR TITLE-ABS-KEY("antiretroviral therapy, highly active")) NOT (TITLE-ABS-KEY("HAART") OR TITLE-ABS-KEY("antiretroviral therapy, highly active")))
EMBASE	(('HIV'/exp OR 'AIDS'/exp) AND ('oral mucosal lesions'/exp OR 'OML'/exp) AND 'humans'/exp) AND (('HAART'/exp OR 'antiretroviral therapy, highly active'/exp) NOT ('HAART'/exp OR 'antiretroviral therapy, highly active'/exp))
CINAHL	((MH "HIV" OR MH "AIDS") AND (MH "oral mucosal lesions" OR "OML") AND MH "humans") AND ((MH "HAART" OR "antiretroviral therapy, highly active") NOT (MH "HAART" OR "antiretroviral therapy, highly active"))
Cochrane Library	(("HIV" OR "AIDS") AND ("oral mucosal lesions" OR "OML") AND "humans") AND (("HAART" OR "antiretroviral therapy, highly active") NOT ("HAART" OR "antiretroviral therapy, highly active"))
ProQuest	((("HIV" OR "AIDS") AND ("oral mucosal lesions" OR "OML") AND "humans") AND (("HAART" OR "antiretroviral therapy, highly active") NOT ("HAART" OR "antiretroviral therapy, highly active"))
Google Scholar	(("HIV" OR "AIDS") AND ("oral mucosal lesions" OR "OML") AND "humans") AND (("HAART" OR "antiretroviral therapy, highly active") NOT ("HAART" OR "antiretroviral therapy, highly active"))

**Table 2:** Demographic characteristics of the included papers

**Study ID**	**Region assessed**	**Sample size (n)**	**Age (in years)**	**Assessment period (in months)**	**Gender ratio (in terms of male percentage)**
Hamza *et al.* [16]	Tanzania	481	Feb-67	14	31%
Lourenço *et al.* [17]	Brazil	340	38	Unspecified	64
Mthethwa *et al.* [18]	South Africa	203	37 (20 - 70)	11 (mean)	One third
Naidu *et al.* [19]	Nepal	81	32.493	Unspecified	66.7
Nittayananta *et al.* [20]	Thailand	157	20 - 60 years	36	45.2
Patil *et al.* [21]	India	100	35.16	Unspecified	56
Rai *et al.* [22]	India	163	32.56 ± 7.69	5	Unspecified
Shu *et al.* [23]	China	1812	Unspecified	>3	68.9
Tappuni *et al.* [24]	UK	231	34	Unspecified	Unspecified

**Table 3:** Comparison between HAART and non-HAART individuals in terms of observed OMLs in the included papers

**Study ID**	**OML Assessment**	**Groups Assessed**	**Key Findings**
Hamza *et al.* [16]	WHO clinical staging criteria	HAART (n=276) vs. non-HAART (n=205)	Non-HAART had a higher risk of OMLs. Oral candidiasis: 0% (HAART) vs. 20.7% (non-HAART). Enlarged parotid gland: 18.2% (HAART) vs. 20.7% (non-HAART).
Lourenço *et al.* [17]	E.C. Clearing house	HAART (n=271) vs. non-HAART (n=69)	OML prevalence: 27.6% (HAART) vs. 55.07% (non-HAART) (p<0.001). Most common OML: Pseudomembranous candidiasis.
Mthethwa *et al.* [18]	E.C. Clearing house	HAART (n=140) vs. non-HAART (n=63)	OML prevalence: 21.4% (HAART) vs. 54% (non-HAART) (p<0.001). No hairy leukoplakia in HAART group.
Naidu *et al.* [19]	WHO	HAART (n=28) vs. non-HAART (n=53)	Higher occurrence of multiple OMLs in HAART (39.6%) vs. non-HAART (35.7%). Oral candidiasis more common in non-HAART group.
Nittayananta *et al.* [20]	E.C. Clearing house	HAART (n=99) vs. non-HAART (n=58)	OMLs: 52% (non-HAART) vs. 57% (HAART long-term). Hyperpigmentation was the most common OML.
Patil *et al.* [21]	E.C. Clearing house	HAART (n=50) vs. non-HAART (n=50)	OML prevalence: 32% (HAART) vs. 56% (non-HAART). Most common OML: Hyperpigmentation.
Rai *et al.* [22]	WHO (2013), E.C. Clearing house	HAART (n=109) vs. non-HAART (n=44)	Lower OML prevalence in HAART group. Erythematous candidiasis and pseudomembranous candidiasis significantly higher in non-HAART group.
Shu *et al.* [23]	WHO	HAART (n=422) vs. non-HAART (n=480)	OML prevalence: Higher in non-HAART. Candida erythematous: 8.87% (HAART) vs. 15.27% (non-HAART) (p=0.001).
Tappuni *et al.* [24]	Not specified	HAART (n=89) vs. non-HAART (n=195)	OML prevalence: 11.4% (HAART) vs. 25.8% (controls). EC most common in both groups.

**Table 4:** Risk of bias of included studies

**Study ID**	**Is the case design adequate**	**Selection**			**Comparability of cases and controls**	**Outcome**			**Total**
		**Case representativeness**	**Control Selection**	**Definition of controls**		**Ascertainment of exposure**	**Same method of ascertainment**	**Non-response**	
Arma *et al.* [18]	*	*	-	-	-	*	*	*	5
Folayan *et al.* [19]	*	*	-	-	-	*	*	*	5
Kamat *et al.* [20]	*	*	-	-	-	*	*	*	5
Ma *et al.* [21]	*	*	-	-	-	*	*	*	5
Tohidinik *et al.* [22]	*	*	-	-	-	**	*	*	6
